# Supplementary material for: Defining neutralization and allostery by antibodies against COVID-19 variants
Source: Nat Commun. 2023 Nov 1;14:6967. doi: 10.1038/s41467-023-42408-x (PMC10618280; doi:10.1038/s41467-023-42408-x)
Supplement: Supplementary file 1 — Supplementary Information [file 41467_2023_42408_MOESM1_ESM.docx]

**Defining neutralization and allostery by antibodies against COVID-19 variants**

Nikhil Kumar Tulsian^*1,2,12^, Palur Venkata Raghuvamsi^1,3,12^, Xinlei Qian^4,12^, Gu Yue^4,5^, Bhuvaneshwari D/O Shunmuganathan^4,5^, Firdaus Samsudin^3^, Wong Yee Hwa^6,7^, Lin Jianqing^6,7^, Kiren Purushotorman^4,5^, Mary McQueen Kozma^4^, Bei Wang^8^, Julien Lescar^6,7^, Cheng-I Wang^8^, Ravindra Kumar Gupta^5,9,10^, Peter John Bond^1,3*^, Paul Anthony MacAry^5,11*^

**Affiliations**

^1^ Department of Biological Sciences, National University of Singapore; Singapore - 117543

^2^ Department of Biochemistry, National University of Singapore; Singapore - 117546

^3^ Bioinformatics Institute, Agency for Science, Technology, and Research (A*STAR); Singapore - 138761

^4^ ­Antibody Engineering Programme, Life Sciences Institute, National University of Singapore; Singapore – 117546

^5^ ­­ Department of Microbiology and Immunology, Yong Loo Lin School of Medicine, National University of Singapore; Singapore -117546

^6^ School of Biological Sciences, Nanyang Technological University; Singapore - 637551

^7^ NTU Institute of Structural Biology; Experimental Medicine Building, Singapore - 636921

^8^ Singapore Immunology Network, Agency for Science, Technology and Research (A*STAR); Singapore – 138648

^9^ Cambridge Institute of Therapeutic Immunology & Infectious Disease (CITIID), Cambridge, UK;

^10^ Department of Medicine, University of Cambridge, Cambridge, UK.

^11^ Life Sciences Institute, National University of Singapore, Singapore 117546, Singapore

^12^ These authors contributed equally.

^*^Correspondence: nikhilkt.science@gmail.com; [peterjb@bii.a-star.edu.sg](mailto:peterjb@bii.a-star.edu.sg); [micpam@nus.edu.sg](mailto:micpam@nus.edu.sg)

**SUPPLEMENTARY DATA**


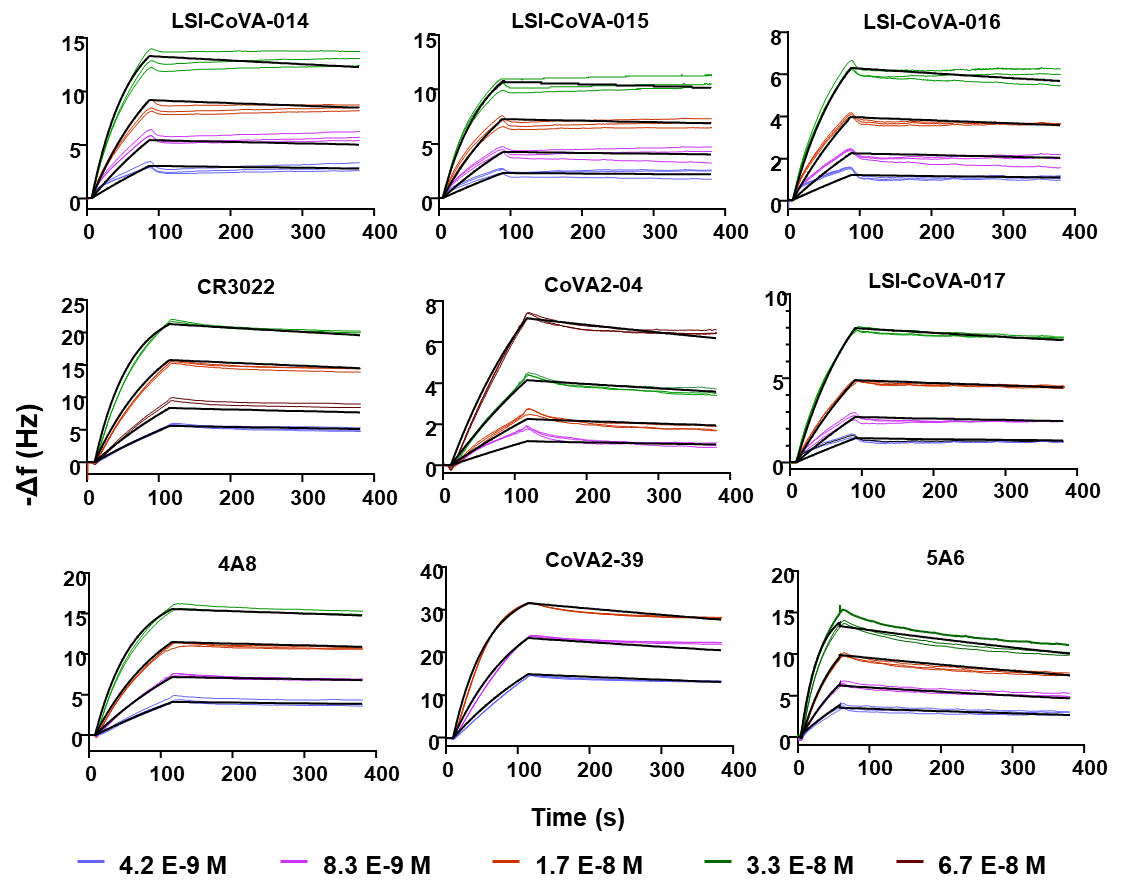


**Supplementary Figure 1: QCM kinetic evaluation of the interactions between Spike and SARS CoV-2 antibodies**

(a) Kinetic plots showing association-dissociation of in-house (LSI-CoVA-014, LSI-CoVA-015, LSI-CoVA-016, and LSI-CoVA-017) and previously studied (CR3022, CoVA2-04, 4A8, CoVA2-39, and 5A6) HuMAbs. Antibodies at varying concentrations (4.2 nM – 66.7 nM) were flowed over Spike (HexaPro, purified from insect cell culture) trimer immobilized onto the surface of a vibrating quartz crystal. Each experiment was performed in 3 independent experiments, indicated by 3 traces for each plot. Thick black lines show the theoretical 1:1 fit obtained using TraceDrawer v1.9.1 evaluation software (Ridgeview Instruments).

**
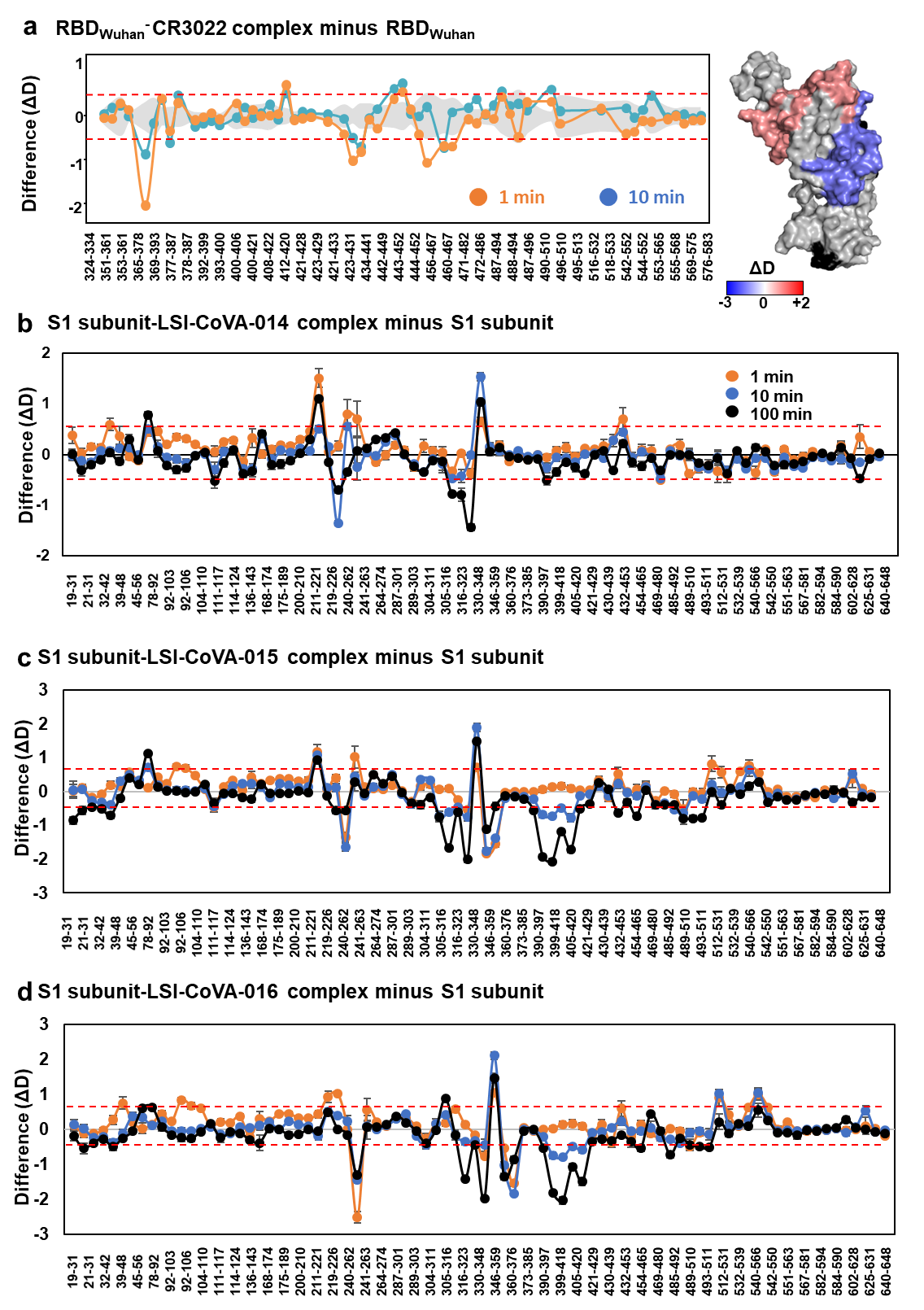
Supplementary Figure 2: Probing conformational changes across the S1 subunit and the RBD-binding antibodies upon their complex formation**

(a) Plot of differences in deuterium exchange between CR3022 antibody-bound and free states of isolated RBD (Wuhan-Hu-1 strain) are shown for indicated labeling times. Residue numbers are indicated for the peptides. Average values (n = 3 independent experiments) were used to generate the plots (Microsoft Excel) with standard deviations indicated as grey shaded area. ± 0.5 Da was considered as a significance threshold, indicated red dashed line. (right panel) Differences at 1 min labeling time are mapped onto high resolution structure of RBD. (b-d) Plots of differences in deuterium exchange between antibody-bound and free states of the S2 subunit of Spike trimer complexes with (b) LSI-CoVA-014, (c) LSI-CoVA-015, and (d) LSI-CoVA-016 are shown for indicated labeling times. Residue numbers are indicated for the peptides, as per supplementary information. Data is represented as mean (n = 6 independent experiments, 2 biological replicates each with technical triplicates) with the standard deviations indicated as error bars. A value of ± 0.5 Da was considered significant, and is indicated by red-dashed line. Statistical analyses were carried out using Deuteros 2.0. Data are provided in Supplementary Information.

**
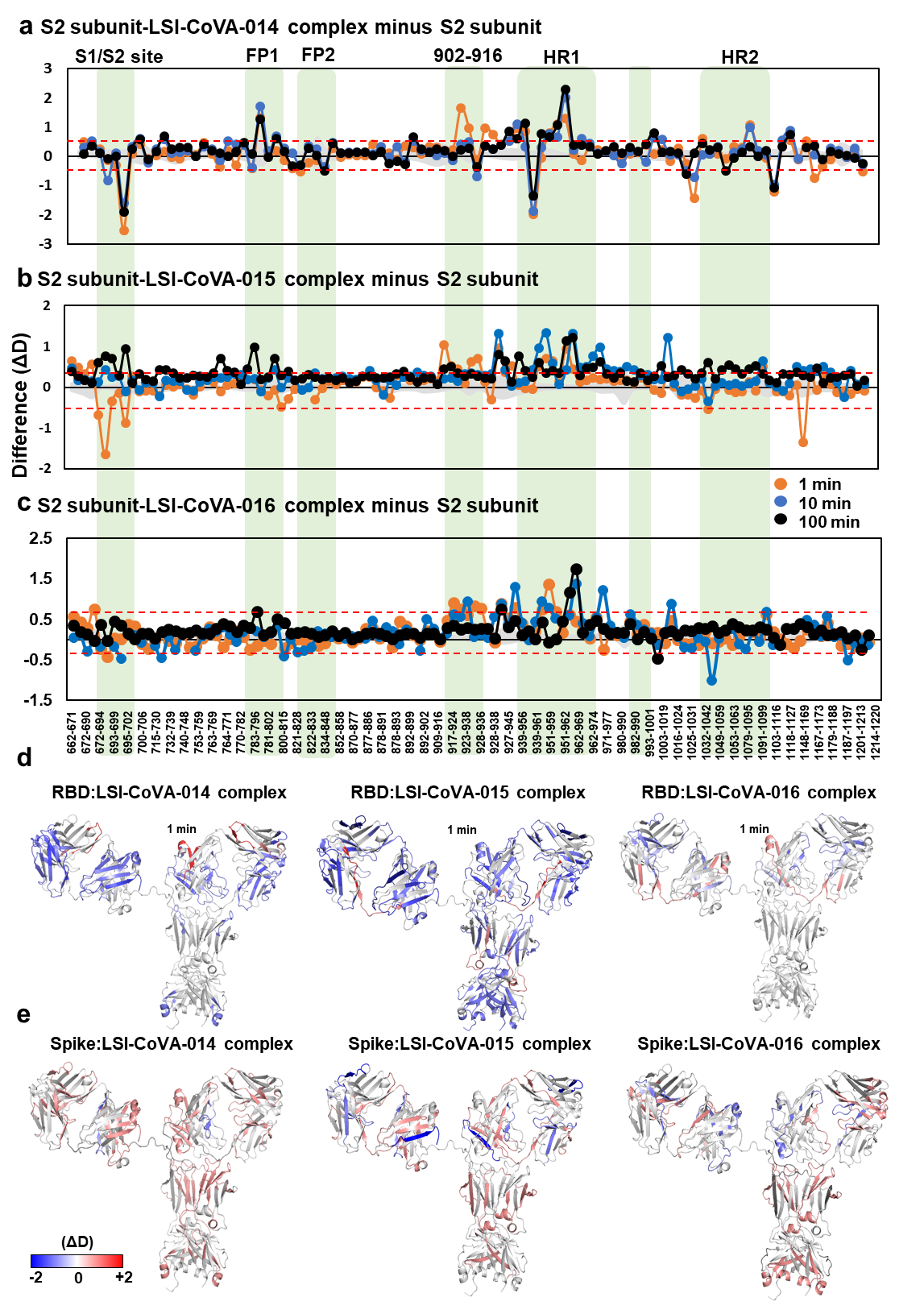
Supplementary Figure 3: Probing conformational changes across the S2 subunit and the RBD-binding antibodies upon their complex formation**

Plots of differences in deuterium exchange between antibody-bound and free states of the S2 subunit of Spike trimer (purified from insect cell culture) complexes with (a) LSI-CoVA-014, (b) LSI-CoVA-015, and (c) LSI-CoVA-016 are shown for indicated labeling times. Residue numbers are indicated for the peptides, as per supplementary information. S1/S2 cleavage site, Fusion peptide (FP) 1 and 2, Heptad Repeats (HR) 1 and 2, Central helix are highlighted in green. Data is represented as mean (n = 6 independent experiments, 2 biological replicates each with technical triplicates) with the standard deviations indicates as grey shaded area. A value of ± 0.5 Da was considered significant, and is indicated by red-dashed line. Statistical analyses were carried out using Deuteros 2.0. This is summarized in HDX Summary Table. Conformational dynamics induced by (d) isolated RBD, and (e) Spike trimer on the three antibodies (left) LSI-CoVA-014, (centre) LSI-CoVA-015, and (right) LSI-CoVA-016 are mapped onto a model of IgG structure, shown in cartoon. Changes in deuterium exchange at the paratope-sites of light (CDRL1-L3) and heavy (CDRH1-H3) chains of each antibody are tabulated in Supplementary Table 2. Data are provided in Supplementary Information.


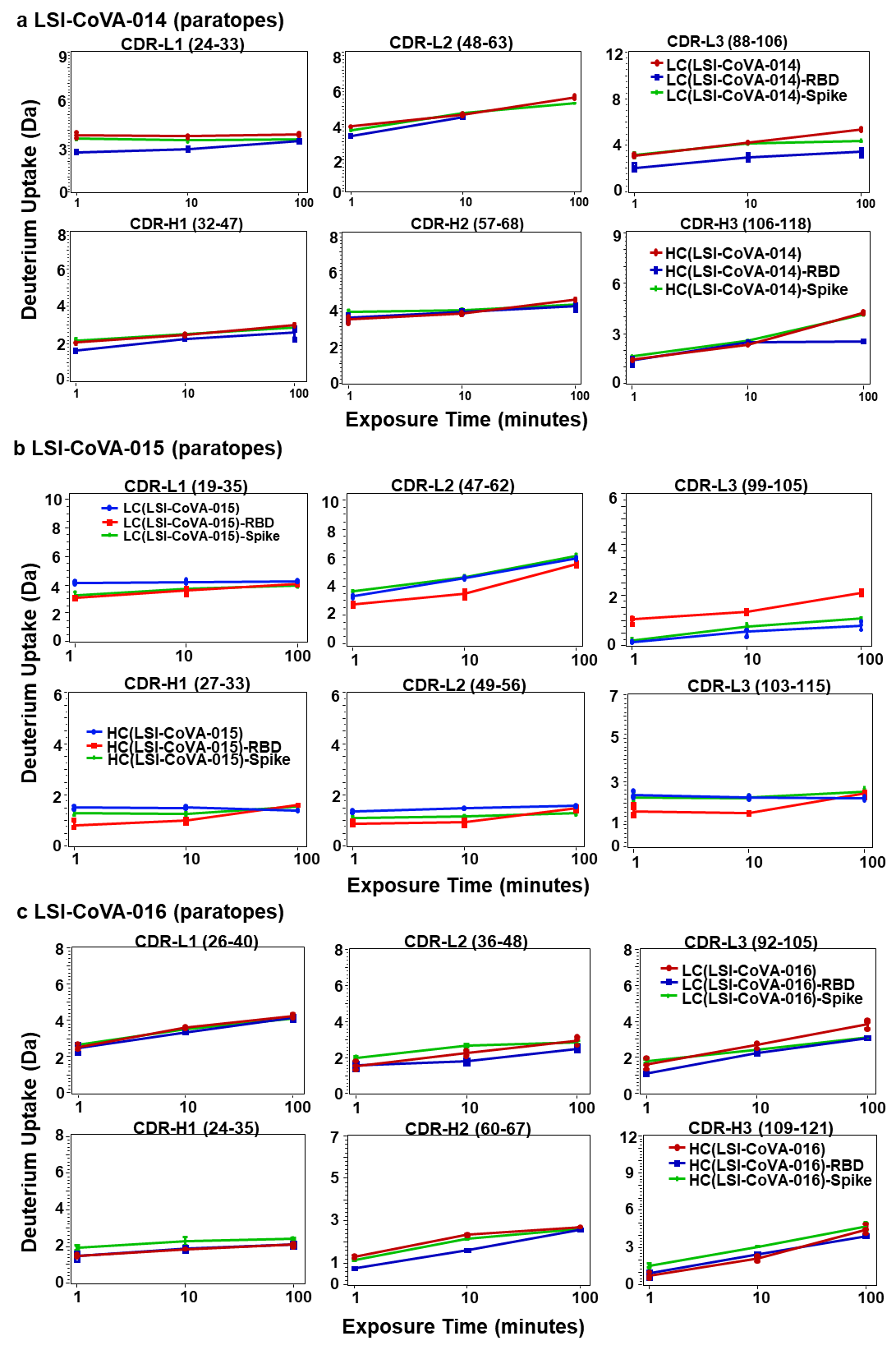


**Supplementary Figure 4: Determining the paratope sites of cryptic site binding antibodies**

Kinetic plots of deuterium uptake (y-axis) at various exposure times (x-axis) for cryptic site binding anti-RBD antibodies (a) LSI-CoVA-014, (b) LSI-CoVA-015, and (c) LSI-CoVA-016 across the three CDR loops of light (top panels) and heavy (bottom panels) chains. Average deuterium uptake values (n = 3 independent experiments) with error bars (estimated as standard deviations) were used to generate the deuterium uptake plots for light chain (LC) and heavy chain (HC) of the three antibodies in the absence and presence of isolated RBD_Wuhan_ and Spike_Wuhan_. Plots were generated using DynamX v3.0 (Waters Inc., USA), with values tabulated in Supplementary Table 2.


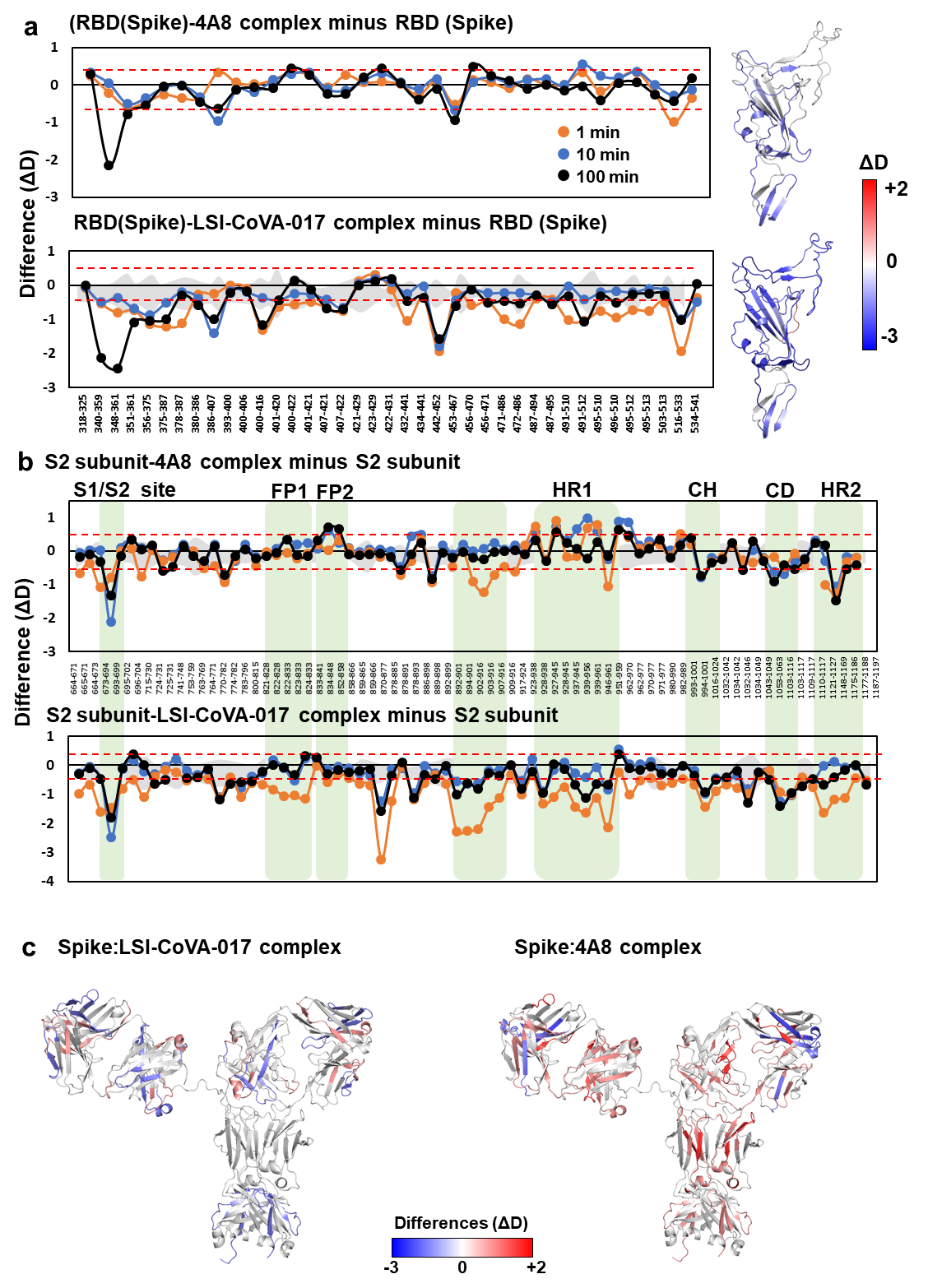


**Supplementary Figure 5: Determining the effect of NTD-binding antibodies on Spike**

Plots showing differences in deuterium exchange across (a) RBD, and (b) the S2 subunit of Spike trimer (purified from insect cell culture) bound to (top panels) 4A8 and (bottom panels) LSI-CoVA-017 antibodies as compared to antibody-free states, across three deuterium labeling time points is indicated. Each dot represents a peptide with their residue numbers indicated along X-axis. The residue numbers of individual peptides for are shown (a) below and (b) between the respective two panels, as per supplementary information. Green boxes highlight important regions, as described previously. Data are provided in Supplementary Information. (Right panels) Differences in deuterium exchange values at 1 min labeling timepoint were mapped onto structures of Spike, with close-up views of RBD shown for clarity. Data is represented as mean (n = 6 independent experiments, 2 biological replicates each with technical triplicates) with the standard deviations indicates as grey shaded area. A value of ± 0.5 Da was considered significant, and is indicated by red-dashed line. Statistical analyses were carried out using Deuteros 2.0. This is summarized in HDX Summary Table. (c) Cartoon representation of a model of antibody showing the differences in deuterium exchange between Spike-bound and free states of the NTD-binding antibody (left) LSI-CoVA-017 (novel antibody in this study), in comparison with (right) 4A8 antibody (previously characterized) at 1 min labeling. Changes in deuterium exchange at the paratope-sites of light (CDRL1-L3) and heavy (CDRH1-H3) chains of each antibody are tabulated in Supplementary Table S2.


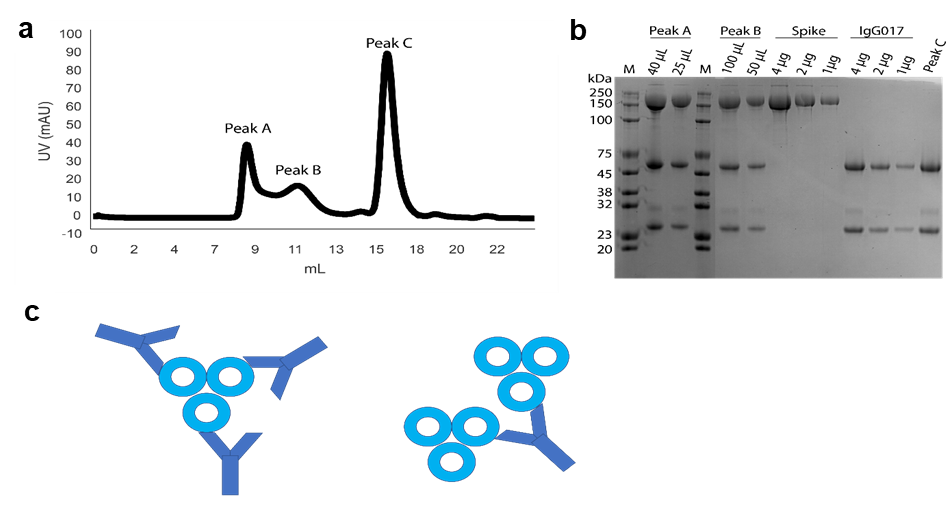


**Supplementary Figure 6: Determining binding stoichiometry of LSI-CoVA-017 with Spike Hexapro trimer**

(a) Chromatogram of mammalian cell culture purified Spike-LSI-CoVA-017 mixture following injection on a Superose 6 Increase 10/300 GL gel filtration column. Peak A corresponds to high molecular weight oligomers, peak B contains lower molecular weight oligomers of Spike-LSI-CoVA-017 complexes, and peak C corresponds to unbound LSI-CoVA-017 added in excess. (b) Image of denaturing polyacrylamide electrophoretic analysis of peak fractions of A and B. LSI-CoVA-017 and Spike were loaded for reference and for densitometry analysis calibration. (c) Schematics of the two most plausible binding modes of interaction between LSI-CoVA-017 (Y-shaped) and Spike trimer (circles), based on the stoichiometric ratios determined and listed in Supplementary Table 3. Data is representative of 2 independent experiments with similar results. Data are provided in Supplementary Information.


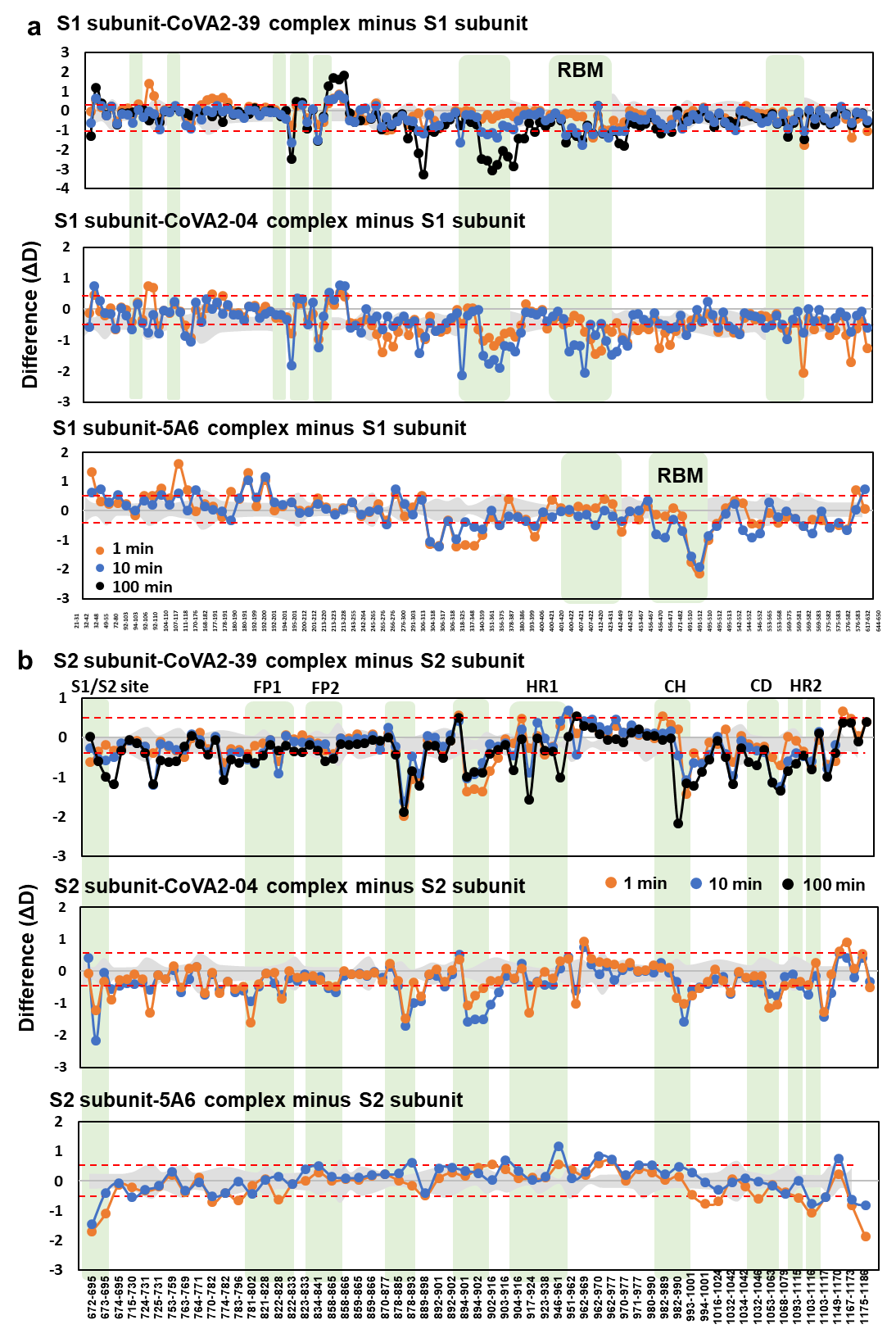


**Supplementary Figure 7: RBM binding antibodies stabilize the dynamics of Spike trimer**

Plots showing differences in deuterium exchange kinetics of (a) the S1 and (b) the S2 subunits of Spike (purified from insect cell culture) in the presence and absence of (top) CoVA2-39, (middle) CoVA2-04, and (bottom) 5A6 HuMAbs for various peptides indicated by their residue numbers (x-axis) common for the three panels each for (a) S1 and (b) S2 subunits are indicated below the panels. Data is represented as mean (n = 6 independent experiments, 2 biological replicates each with technical triplicates) with the standard deviations indicates as grey shaded area. A value of ± 0.5 Da was considered significant, and is indicated by red-dashed line. Statistical analyses were carried out using Deuteros 2.0. Green boxes highlight the epitope sites spanning RBM, and various domains of the S2 subunit as indicated. Data are provided in Supplementary Information.


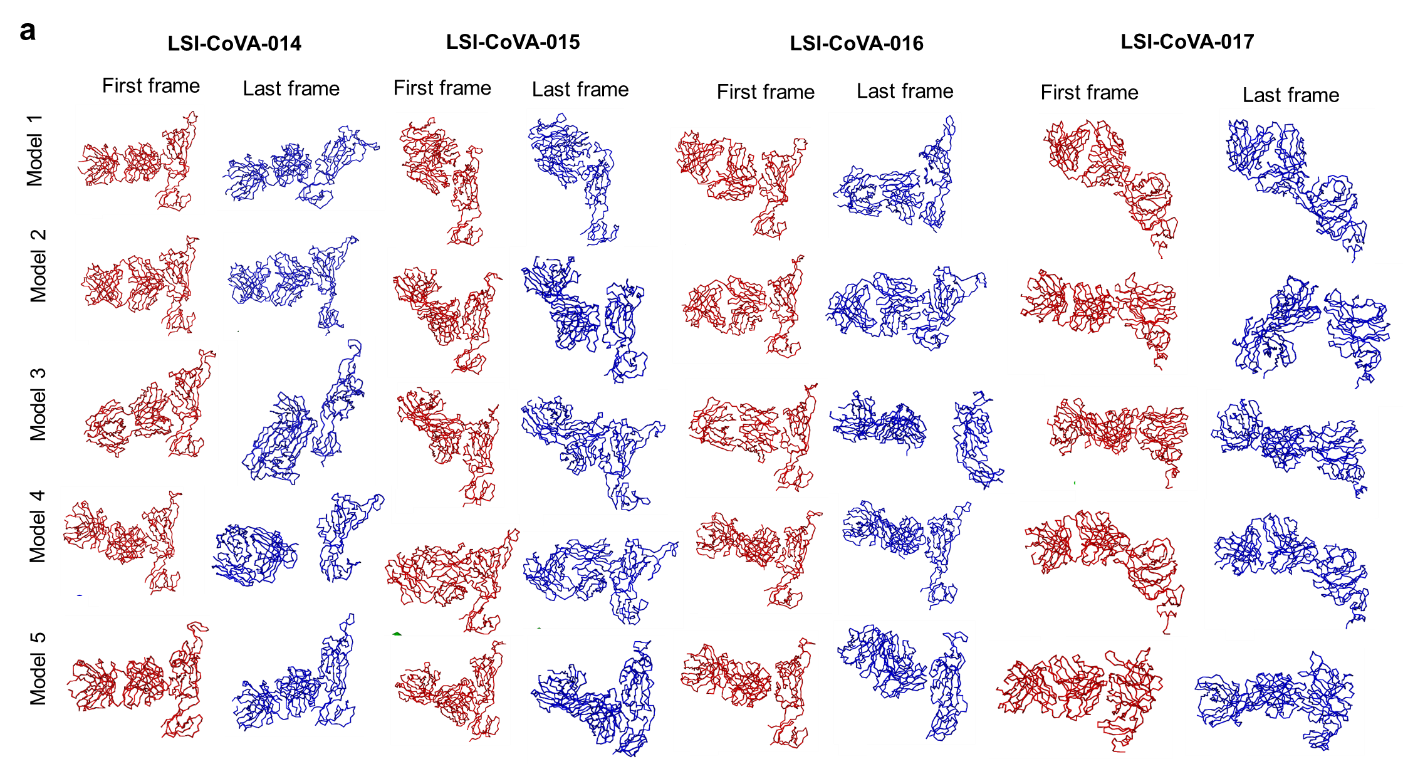


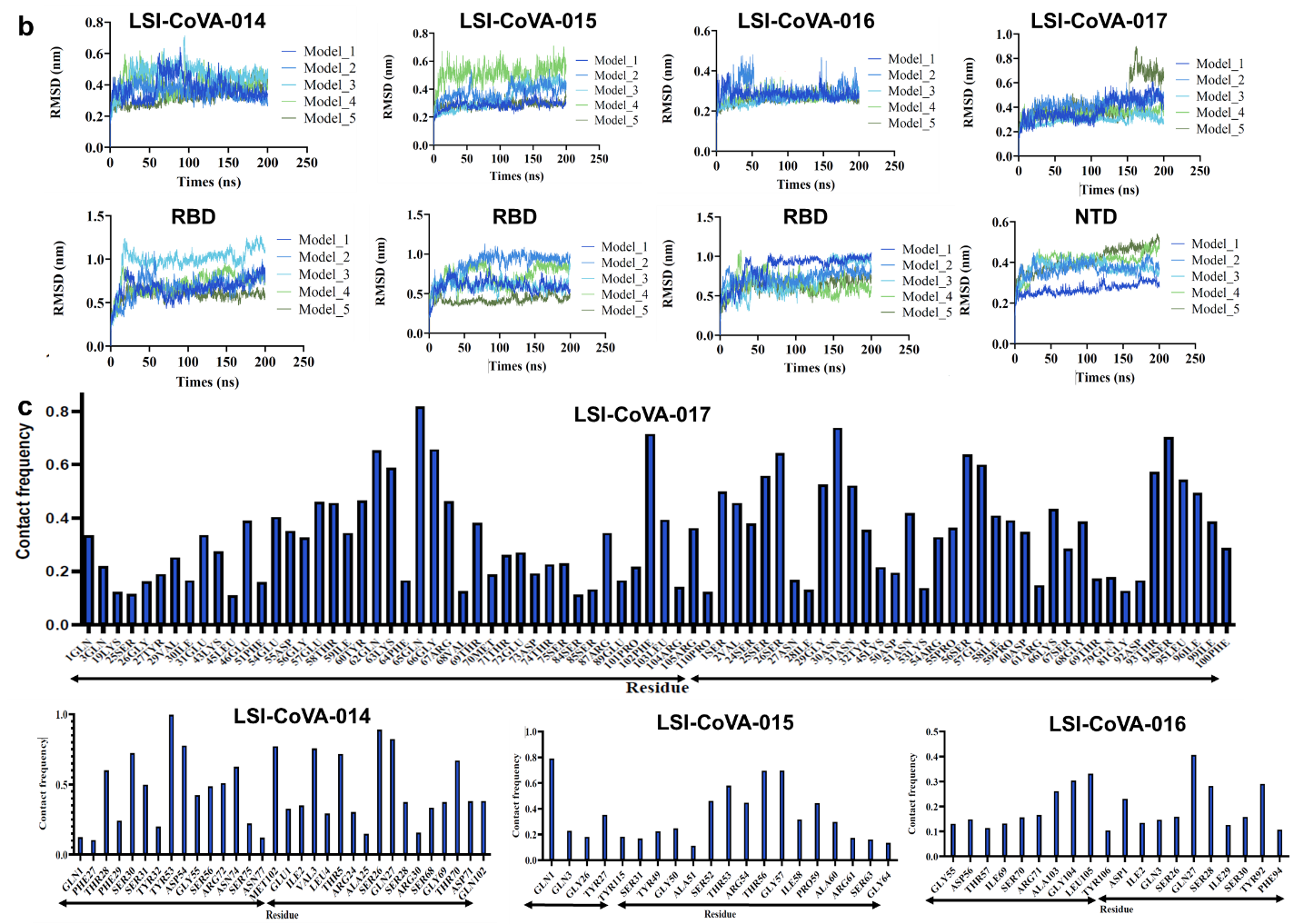


**Supplementary Figure 8: Visualization and cluster analysis of models of Fab:RBD/NTD complexes**

(a) Initial (red) and final (blue) trace representations of (i) RBD-LSI-CoVA-014, (ii) RBD-LSI-CoVA-015, (iii) RBD-LSI-CoVA-016, and (iv) NTD-LSI-CoVA-017 antigen-antibody (Fab) complexes from 200 ns simulations. (b) Variations in RMSD of RBD and NTD and Fab after least-squares fit to the backbone of the complexes for five models from the four systems for 200 ns simulations. (c) Contact frequencies (Y-axis) greater than 0.1 (residues interacting with glycan for at least 10% of simulation time) of the Fab residues (X-axis) from the four antibodies with the glycan moiety of RBD or NTD during 200 ns simulations. Models wherein Fab separates from RBD or NTD were not considered and the maximum contact frequency value of each residue from remaining model trajectories were plotted. Double head arrow indicate the heavy and light chain residues along X-axis respectively. Data are provided in Supplementary Information.

**
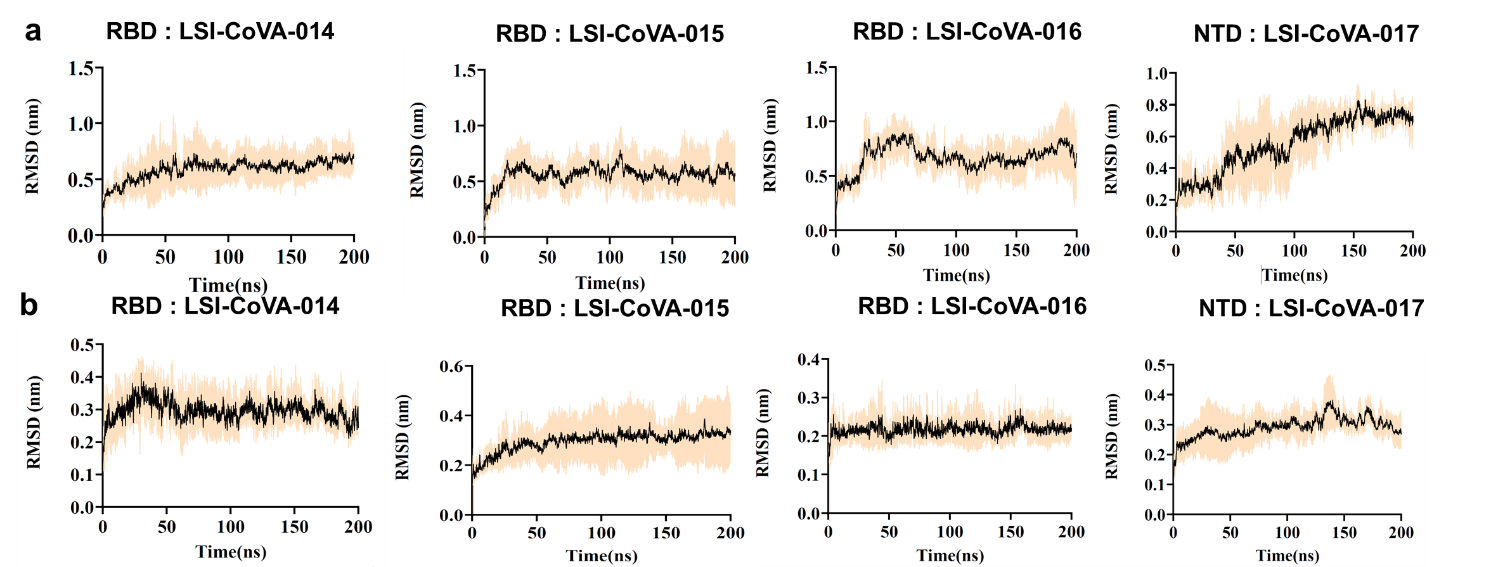
**

**
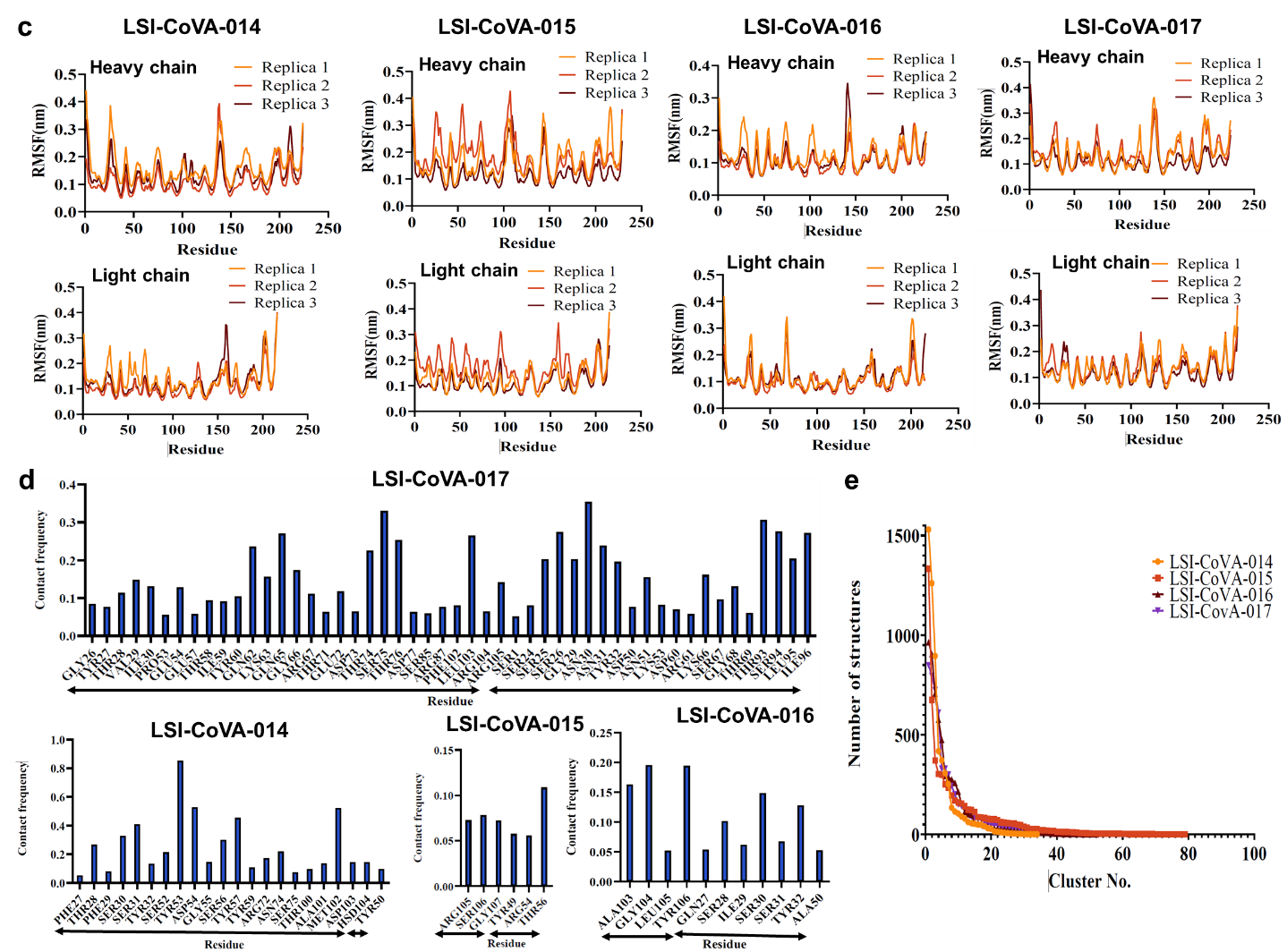
**

**Supplementary Figure 9: Determining the variations of simulations between different Fab:RBD/NTD** **models and the role of glycans**

(a) Plots showing average RMSD of RBD and NTD, after least-squares fit to the backbone of the complexes for the selected pose from each of 200ns long triplicate RBD/NTD:Fab complex simulations. Average RMSD and standard deviation are shown as a solid black line and orange shade, respectively. (b) Plots showing average RMSD of LSI-CoVA-014, LSI-CoVA-015, LSI-CoVA-016, and LSI-CoVA-017, after least-squares fit to the backbone of the complexes for the selected pose from each of 200ns long triplicate RBD/NTD:Fab complex simulations. Average RMSD and standard deviation are shown as a solid black line and orange shade, respectively. (c) Root mean square fluctuation (RMSF) of triplicate simulations of selected pose of LSI-CoVA-014, LSI-CoVA-015, LSI-CoVA-016, and LSI-CoVA-017 antibodies for heavy and light chains from each model bound to RBD and NTD. (d) Contact frequencies >0.05 (Y-axis) (residues interacting with glycan for at least 5% of simulation time) of the Fab residues (X-axis) from the four antibodies with the glycan moiety of RBD or NTD for selected pose triplicate of simulations of each 200 ns long simulations. Double head arrow indicate the heavy and light chain residues along X-axis respectively. (e) Plot showing the size of each cluster of RBD/NTD with LSI-CoVA-014 (black), LSI-CoVA-015 (red), LSI-CoVA-016 (cyan), and LSI-CoVA-017 (green). Cluster analysis was performed for concatenated trajectories derived from triplicate 200 ns simulations for each complex using GROMOS method with an RMSD cut-off of 0.35 nm. Data are provided in Supplementary Information.


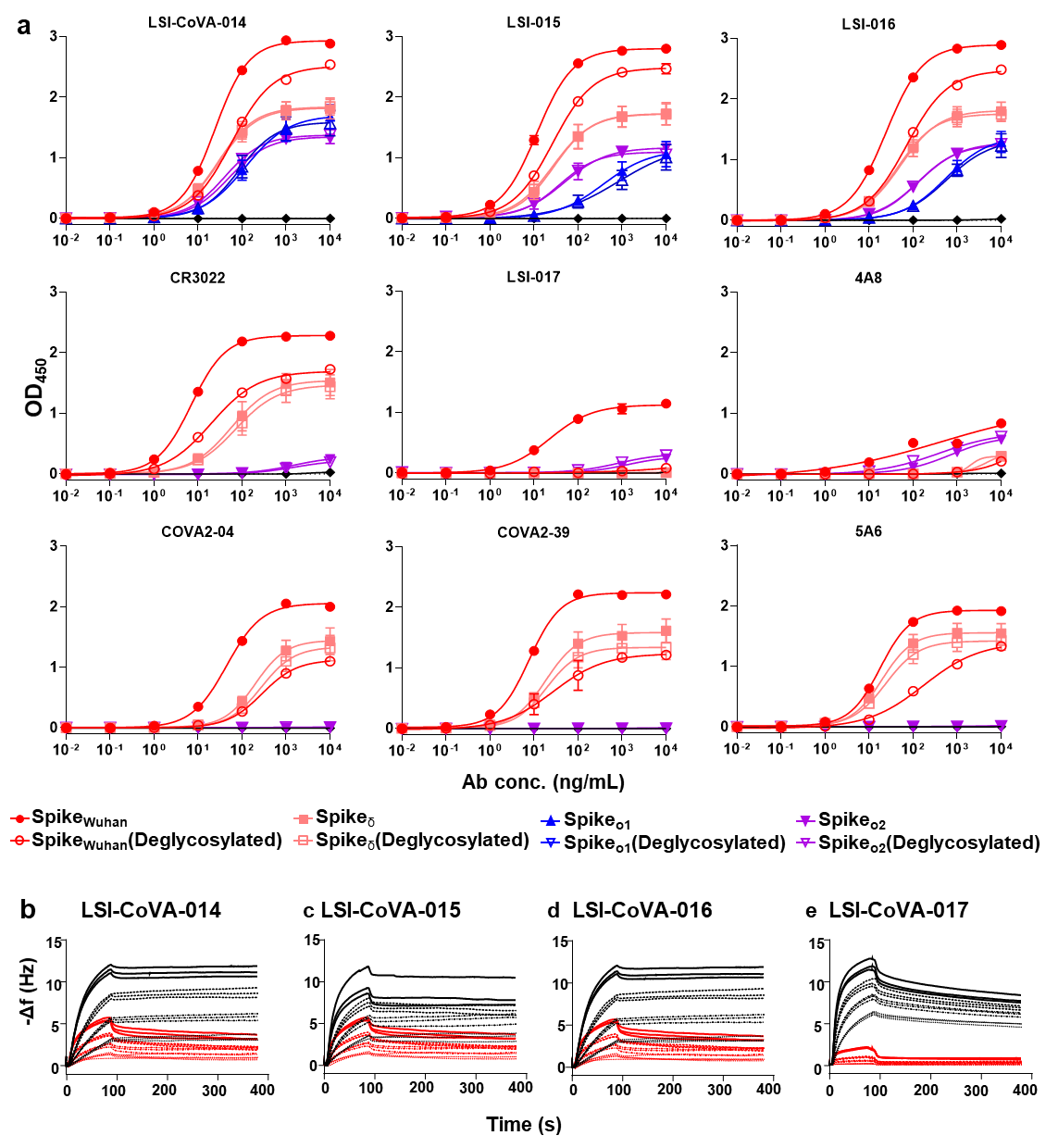


**Supplementary Figure 10: Essential role of glycan groups in facilitating antigen-antibody interactions**

Plots showing varying concentrations of the nine antibodies to glycosylated (closed plots) and deglycosylated (open plots) trimers of Wuhan-Hu-1(Hexapro) (circle plots), Delta (square plots), Omicron BA.1 (blue triangles), and Omicron BA.2 (lilac triangles). Spike trimers used were purified from mammalian cell culture. LSI-CoVA-017 and 4A8 demonstrate no binding activity, with minimal or negligible reduction in binding activity to deglycosylated Spike VoCs. Values (n = 3 independent experiments) were determined by ELISA and the plots were generated using Prism (GraphPad 9.0). Average and standard deviations (mean ± S.E.M.) are shown. (b-e) Antibodies at varying concentrations (4.2 nM – 133 nM) were flowed over Spike (black) and deglycosylated (red) Spike trimer (insect-cell derived) immobilized onto the surface of a vibrating quartz crystal. Data is collected from 3 independent experiments with similar results. Data are provided in Supplementary Information.


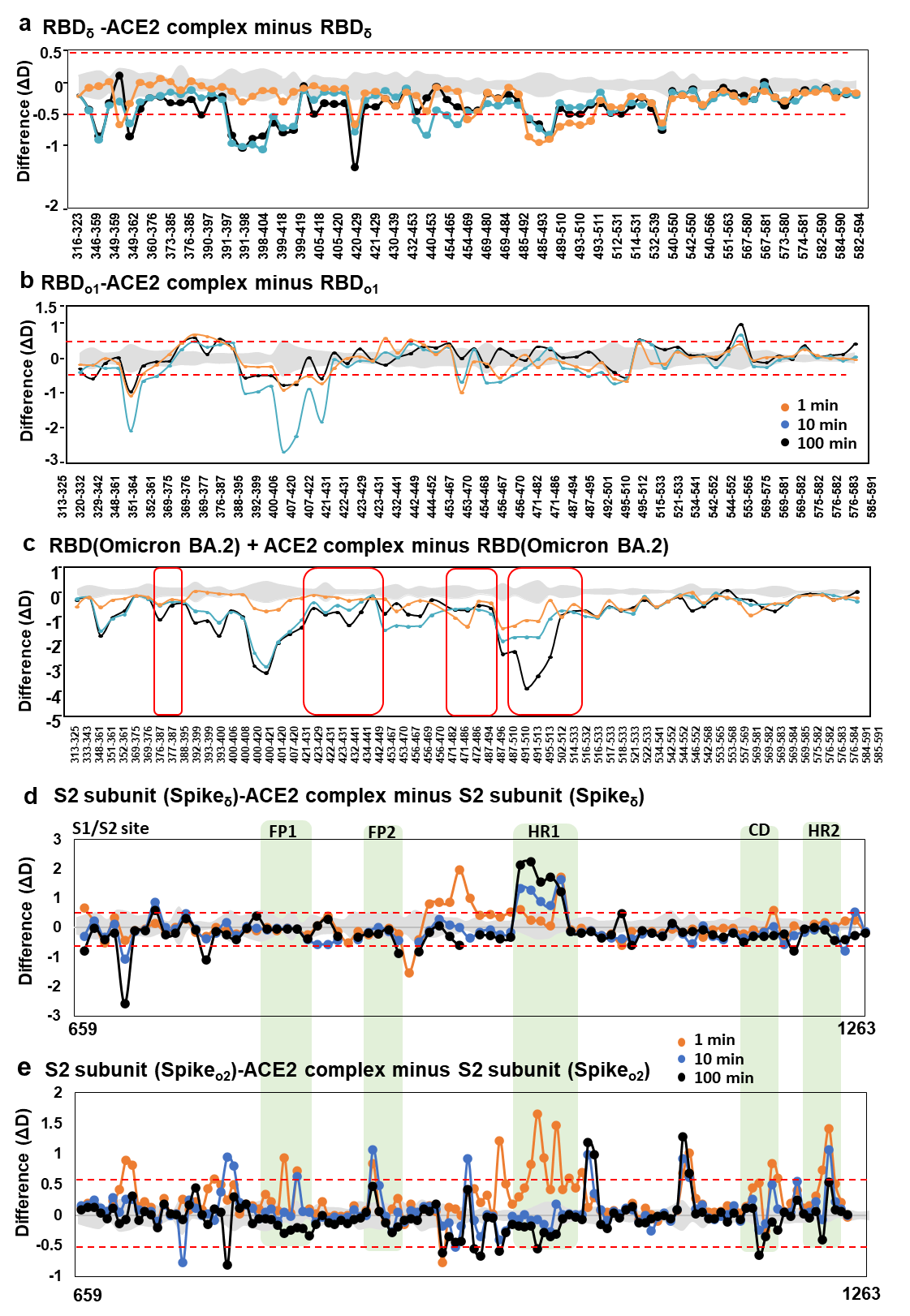


**Supplementary Figure 11. Determining ACE2 binding footprint to RBD Delta and Omicron variants**

Plots showing differences in deuterium exchange between the presence and absence of ACE2 for isolated RBD of (a) Delta, (b) Omicron BA.1, and (c) Omicron BA.2 variants, across indicated labelling times. Pepsin-proteolyzed peptides of the corresponding RBD is represented by dots, with their residue numbers indicated. Data is represented as mean (n = 3 independent experiments) with the standard deviations indicates as grey shaded area. A value of ± 0.5 Da was considered significant, and is indicated by red-dashed line. Statistical analyses were carried out using Deuteros 2.0, and tabulated in Supplementary Table 6. Plots comparing differences in deuterium exchange (ΔD) in the presence and absence of ACE2 for various peptides across the S2 subunits of Spike variants (d) Delta and (e) Omicron BA.2. Various labelling timepoints are indicated with peptide numbers tabulated in supplementary information. Various regions across the S2 subunit showed significant changes in deuterium exchange and are highlighted in green. Data are provided in Supplementary Information.


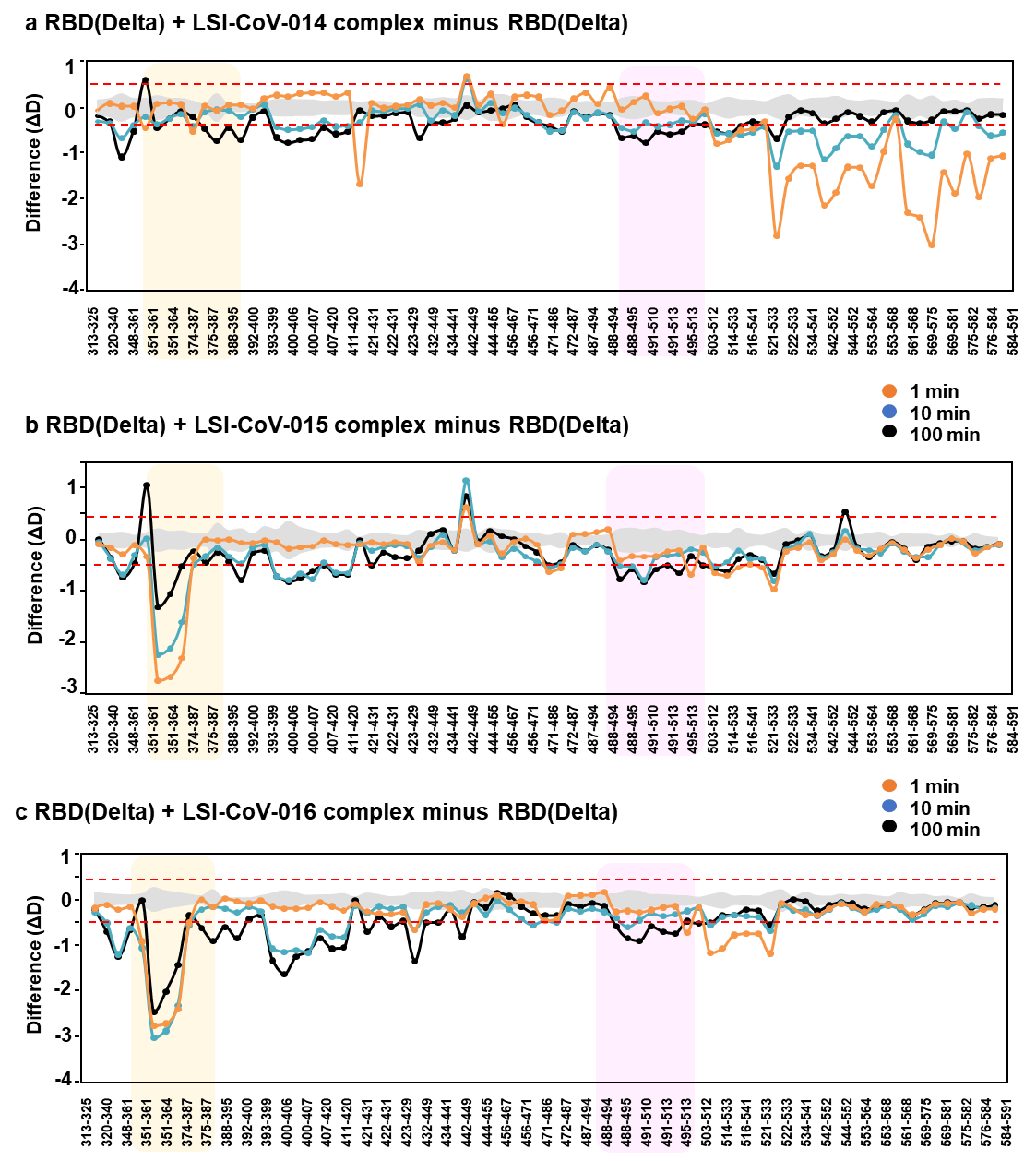


**Supplementary Figure 12. Cryptic site binding antibodies bind weakly to RBD Delta variant**

Plot of differences in deuterium exchange (y-axis) for various pepsin-digested peptides of isolated RBD(delta) in the presence and absence of (a) LSI-CoVA-014, (b) LSI-CoVA-015, and (c) LSI-CoVA-016 antibodies at various labeling times are indicated. Data is represented as mean (n = 3 independent experiments) with the standard deviations indicates as grey shaded area. A value of ± 0.5 Da was considered significant, and is indicated by red-dashed line. Statistical analyses were carried out using Deuteros 2.0. The antibody-binding epitope sites are highlighted in yellow, while ACE2-binding RBM motif are highlighted in pink. Data are provided in Supplementary Information.


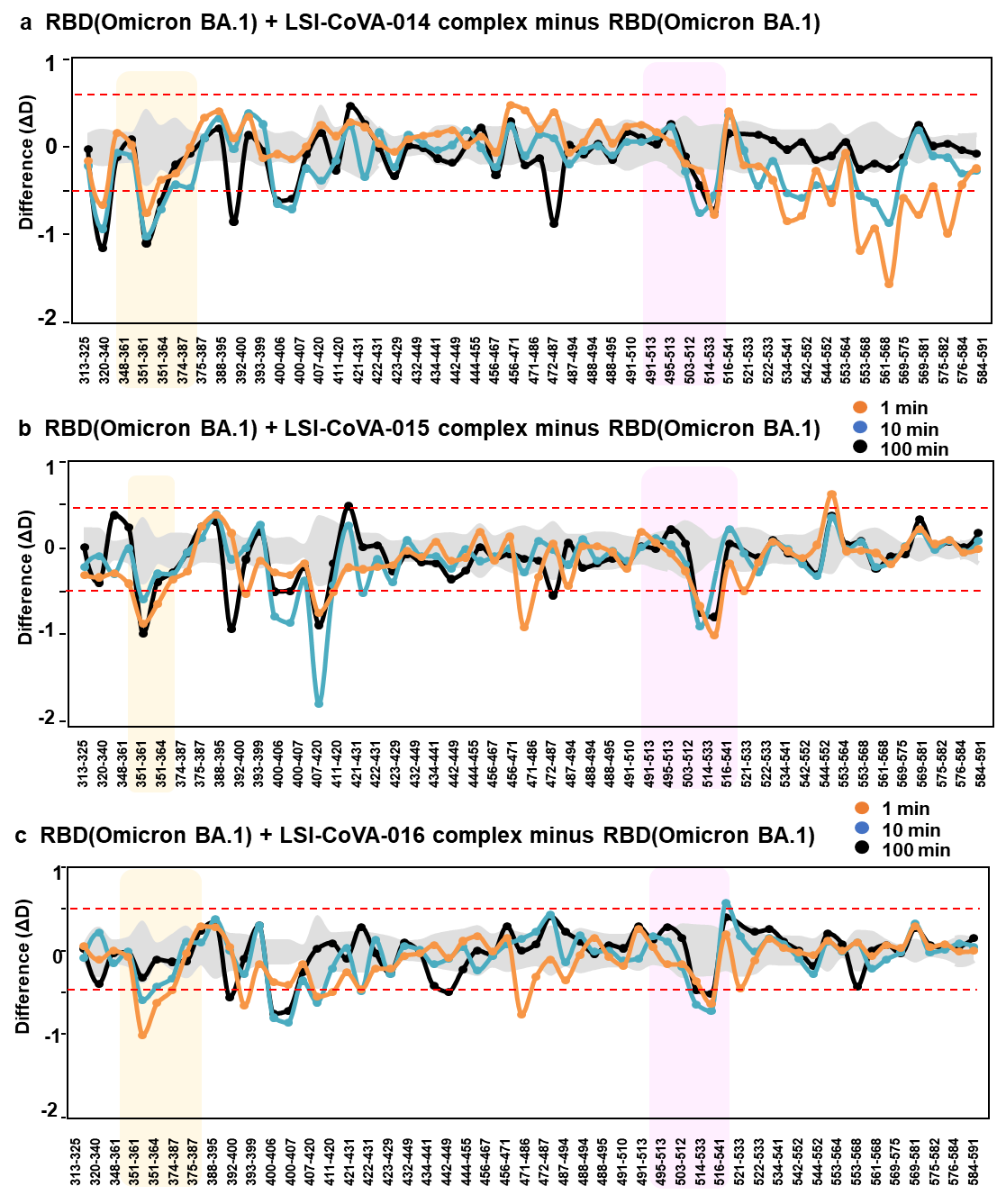


**Supplementary Figure 13. Cryptic site binding antibodies bind weakly to RBD Omicron (BA.1) variant**

Plot of differences in deuterium exchange (y-axis) for various pepsin-digested peptides of isolated RBD Omicron (BA.1) variant in the presence and absence of (a) LSI-CoVA-014, (b) LSI-CoVA-015, and (c) LSI-CoVA-016 antibodies at various labeling times are indicated. Average values (n=3 independent experiments) with standard deviations (grey) shown, with plots generated from DynamX v3.0. ± 0.5 Da was considered as a significance threshold and indicated by red dashed line. The antibody-binding epitope sites are highlighted in yellow, while ACE2-binding RBM motif are highlighted in pink. Data are provided in Supplementary Information.


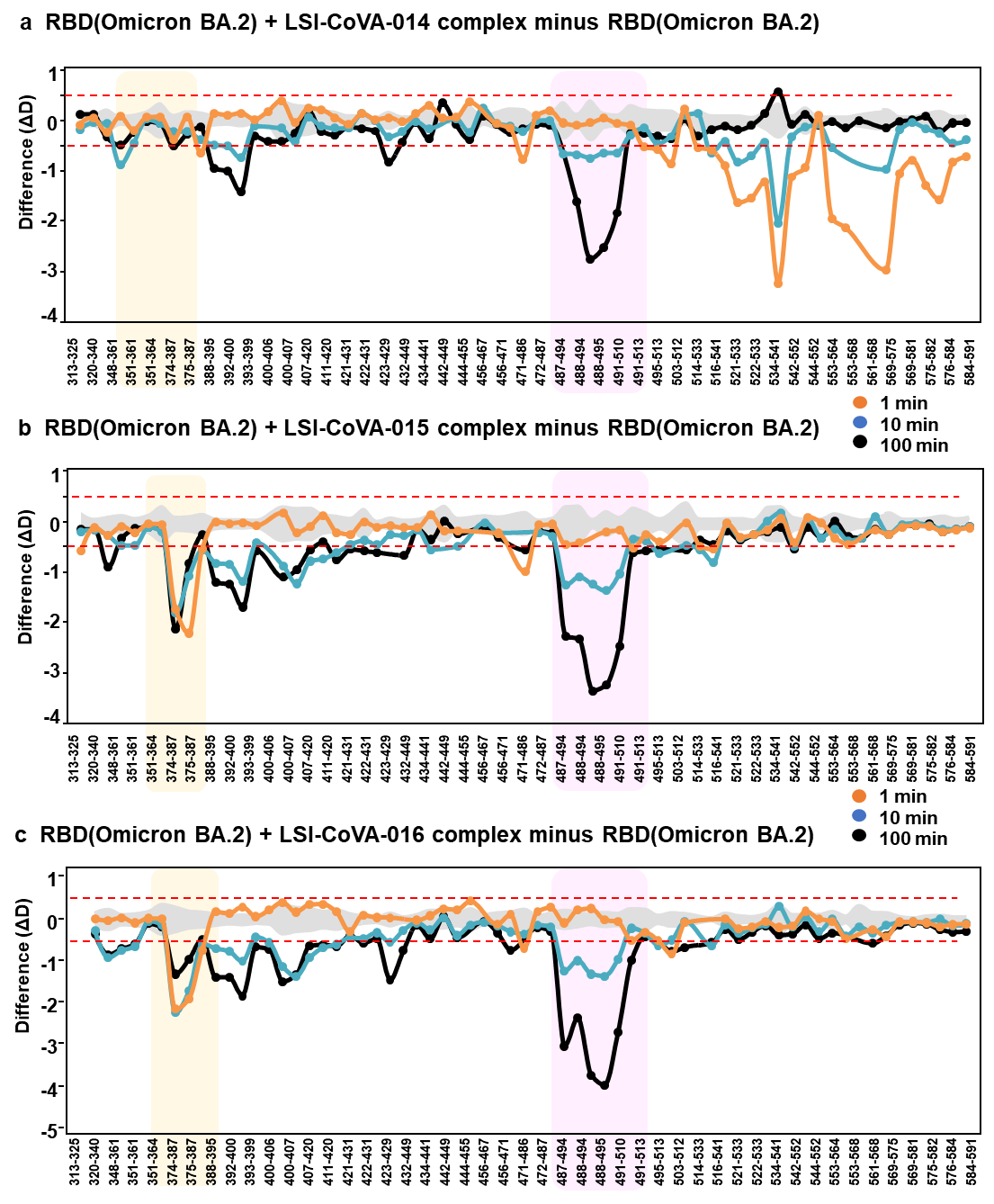


**Supplementary Figure 14. Determining epitope sites of weak-binding antibodies to RBD Omicron (BA.2) variant**

Plot of differences in deuterium exchange (y-axis) for various pepsin-digested peptides of isolated RBD Omicron (BA.2) variant in the presence and absence of (a) LSI-CoVA-014, (b) LSI-CoVA-015, and (c) LSI-CoVA-016 antibodies at various labeling times are indicated. Average values (n = 3 independent experiments) with standard deviations (grey) shown, with plots generated from DynamX v3.0. A value of ± 0.5 Da was considered as a significance threshold and indicated by red dashed line. The antibody-binding epitope sites are highlighted in yellow, while peptide clusters spanning ACE2-binding RBM motif are highlighted in pink. Data are provided in Supplementary Information.

**
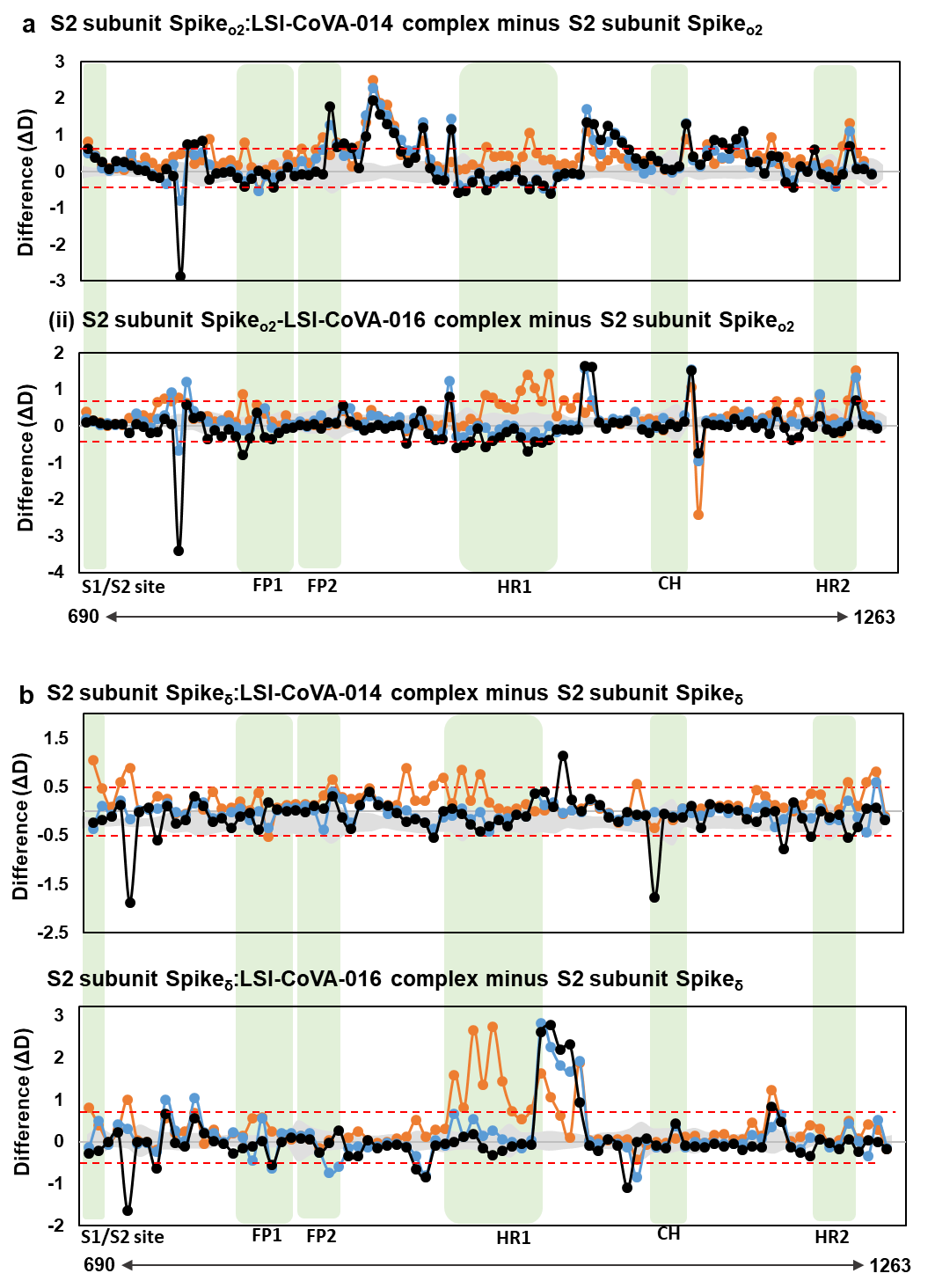
**

**Supplementary Figure 15: Weak neutralizers bind to Spike VoCs at cryptic epitope site**

Plots comparing differences in deuterium exchange across the S2 subunit of (a) Spike_o2_ and (b) Spike_δ_ complexed to (top) LSI-CoVA-014 and (bottom) LSI-CoVA-016 versus the apo states. Peptides spanning the S2 subunit are indicated along the X-axis as N- and C- terminal residue numbers (common to the two plots in each panel). Average (n = 3 independent experiments) values were used to generate the plots, with standard deviations in grey. A value of ± 0.5 Da was considered significant, and is indicated by red-dashed line. Statistical analyses were carried out using Deuteros 2.0. Key regions of the S2 subunit showing distinct deuterium exchange differences are highlighted in green and labeled. Data are provided in Supplementary Information.


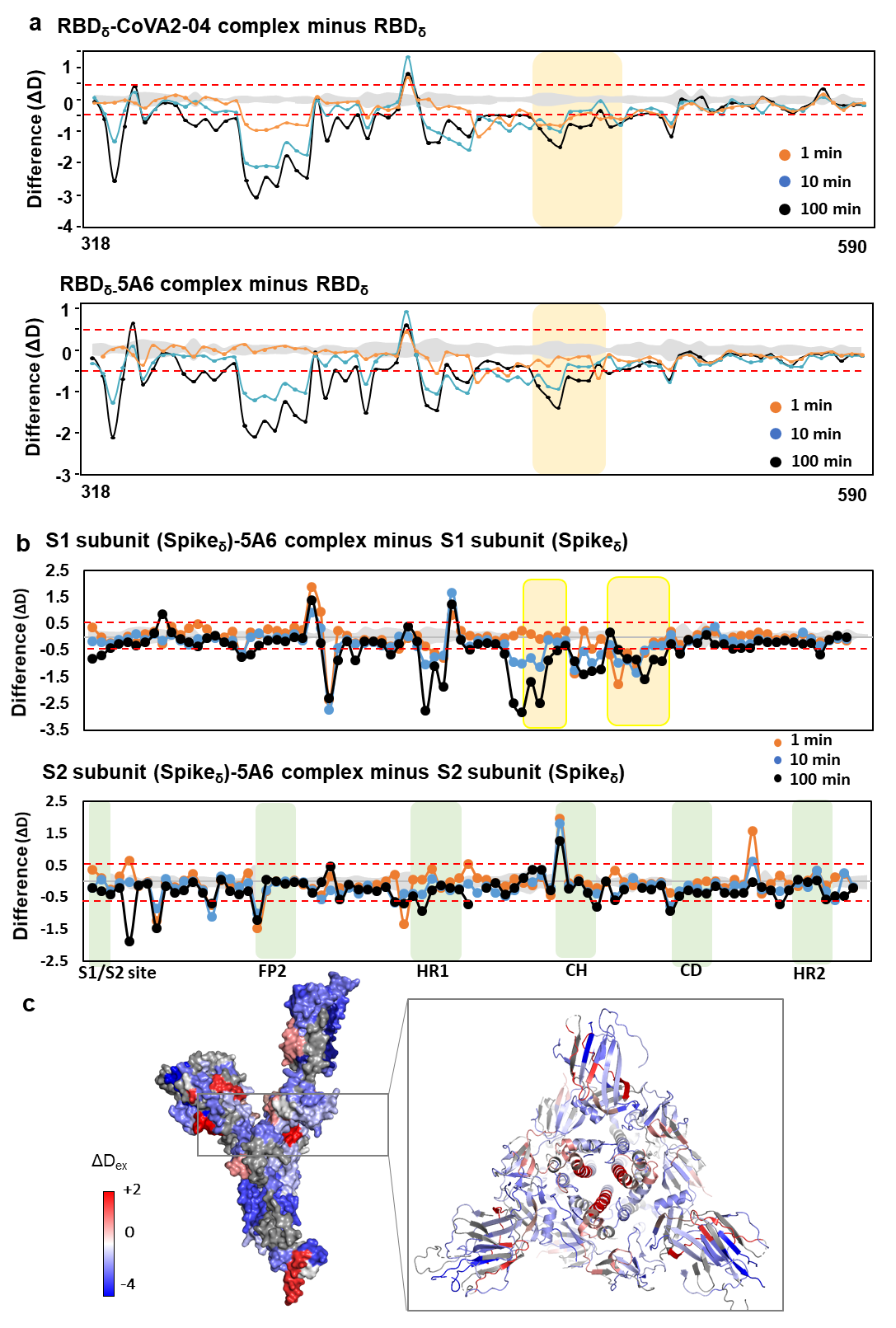


**Supplementary Figure 16. Strong neutralizers show minimal binding to Spike_δ_**

(a) Effects of binding of RBM-binding antibodies (top) CoVA2-04 and (bottom) 5A6 on RBD_δ_ are shown as plots showing differences in deuterium exchange. Antibody binding epitope sites are in yellow, and exhibit minor changes in deuterium exchange, as opposed to large-scale decreased deuterium exchange observed at other peptides. (b) Difference in deuterium exchange in Spike_δ_ trimer upon binding to 5A6 antibody are shown for peptides spanning the (top) S1 and (bottom) S2 subunits. Key regions are highlighted. (c) Differences at 1 min labeling timepoint are mapped onto Spike_δ_ (PDB: 7W98) monomer, shown as surface representation. A cross section across the trimer is shown as inset, which highlights the quaternary effects of 5A6 binding. The N- and C- terminal residue numbers for the peptides (represented by circles in difference plots) are indicated along X-axis and are listed in Supplementary Information. For each plot, data is represented as mean (n = 3 independent experiments) with the standard deviations indicates as grey shaded area. A value of ± 0.5 Da was considered significant, and is indicated by red-dashed line. Statistical analyses were carried out using Deuteros 2.0. Data are provided in Supplementary Information.


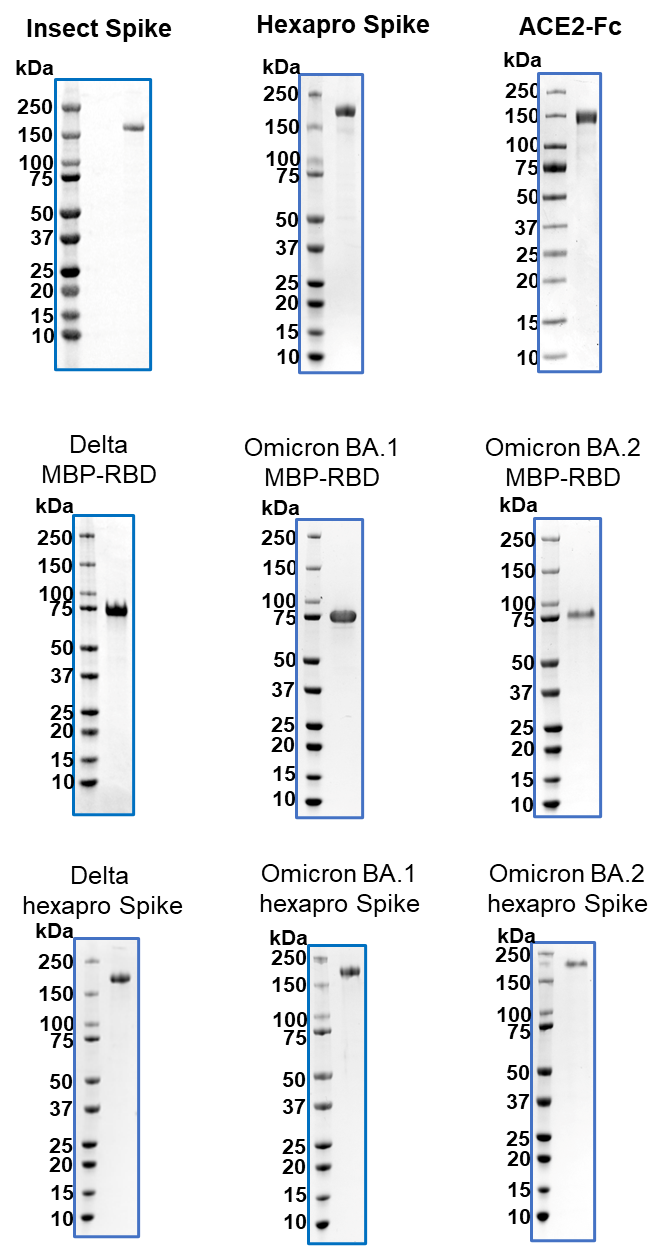


**Supplementary Figure 17. Purification of isolated RBD and Spike constructs**

Purity and integrity of isolated RBD (MBP-tagged) and Spike proteins of Wuhan, Delta, and Omicron variants are shown by denatured polyacrylamide gel electrophoresis images. Left lanes correspond to protein molecular weight ladder, and right lanes show the corresponding protein bands. Data is representative of 2 independent experiments with similar results. Data are provided in Supplementary Information.


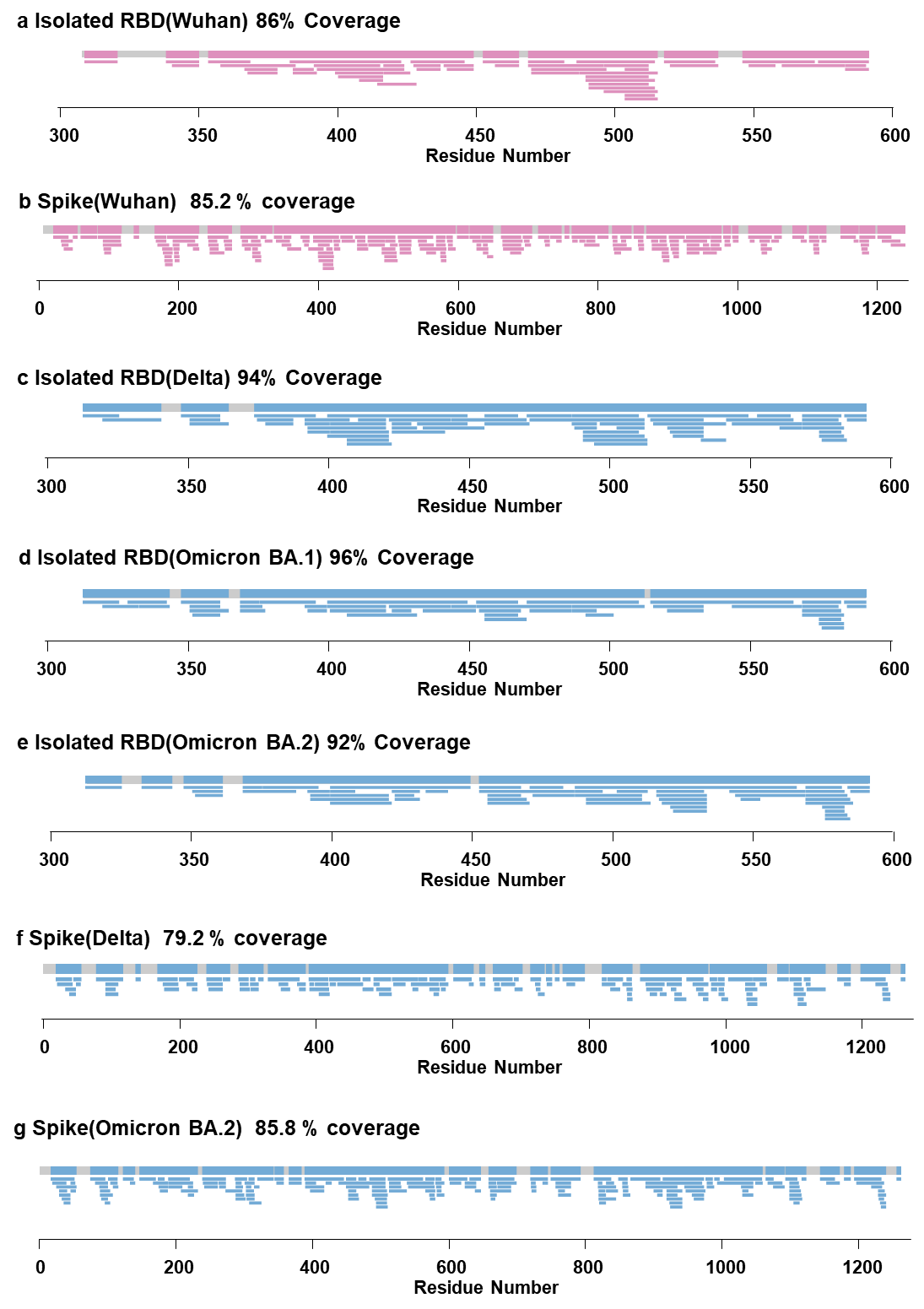


**Supplementary Figure 18. Pepsin-digest map of isolated RBD and Spike**

Sequence coverage maps showing pepsin-digested peptides (indicated by thick lines) encompassing primary sequence for (a) isolated RBD(Wuhan) – 86%, (b) Spike(Wuhan) – 85.2%, (c) isolated RBD (Delta) – 94%, (d) isolated RBD (Omicron BA.1) – 96%, (e) isolated RBD (Omicron BA.2) – 92%, (f) Spike(Delta) – 79.2%, and (g) Spike(Omicron BA.2) – 85.8% are shown. The residue numbers are indicated on x-axis. All the Spike trimers used for HDX were purified from insect cell culture.

**Supplementary Table 1:** Quartz crystal microbalance analysis of antibody binding kinetics to mammalian cell culture purified Hexapro Spike trimer (0.03 μM). k_a_ = association constant; k_d_: dissociation constant.

| **Antibody** | **B_max_** | **k_a_ (1/[(M^-^s)]** | **k_d_ [1/s]** | **K_D_ (M)** | **Chi^2^** |
| --- | --- | --- | --- | --- | --- |
| LSI-CoVA-016 | 9.61 | 3.90 E+05 | 3.00 E-04 | 7.68 E-10 | 0.06 |
| LSI-CoVA-017 | 19.66 | 4.46 E+05 | 7.48 E-05 | 1.67 E-10 | 0.12 |
| 4A8 | 18.03 | 5.74 E+05 | 1.95 E-04 | 3.40 E-10 | 0.10 |
| LSI-CoVA-015 | 13.86 | 5.61 E+05 | 1.88 E-04 | 3.35 E-10 | 0.21 |
| COVA2-39 | 36.07 | 1.25 E+06 | 4.87 E-04 | 3.88 E-10 | 0.57 |
| LSI-CoVA-014 | 17.12 | 5.57 E+05 | 2.88 E-04 | 5.16 E-10 | 0.35 |
| CR3022 | 24.53 | 6.05 E+05 | 3.16 E-04 | 5.21 E-10 | 0.38 |
| COVA2-04 | 15.25 | 9.32 E+04 | 5.55 E-04 | 5.96 E-09 | 0.05 |
| 5A6 | 15.52 | 1.18 E+06 | 8.87 E-04 | 7.55 E-10 | 0.15 |

**Supplementary Table 2:** Differences in deuterium exchange values for complementarity-determining regions (CDR) of light (CDR L1-L3) and heavy (CDR H1-H3) chains of various antibodies in the presence and absence of RBD_iso_ (top) and Spike_Wuhan_ (bottom) (purified from insect cell culture) as indicated. Positive differences (>0.5 D) are highlighted in red, and negative differences (<-0.5 D) are in green.

|  | CDRL1 | CDRL2 | CDRL3 | CDRH1 | CDRH2 | CDRH3 |
| --- | --- | --- | --- | --- | --- | --- |
|  | RBD | RBD | RBD | RBD | RBD | RBD |
| LSI-CoVA-014 | -1.00 | -0.32 | -1.86 | 0.65 | -1.74 | -1.70 |
| LSI-CoVA-015 | -0.51 | -1.73 | -0.70 | -1.08 | -1.18 | 0.41 |
| LSI-CoVA-016 | -0.23 | -0.41 | -0.76 | -0.25 | -0.55 | 0.24 |
| LSI-CoVA-017 | 0.35 | -0.36 | -0.05 | -0.21 | -0.45 | -0.17 |

|  | CDRL1 | CDRL2 | CDRL3 | CDRH1 | CDRH2 | CDRH3 |
| --- | --- | --- | --- | --- | --- | --- |
|  | Spike | Spike | Spike | Spike | Spike | Spike |
| LSI-CoVA-014 | -0.55 | -0.52 | -0.81 | 0.45 | 0.40 | -1.01 |
| LSI-CoVA-015 | 0.17 | -1.88 | -0.41 | -0.89 | -0.27 | 0.60 |
| LSI-CoVA-016 | -0.16 | -0.27 | -0.71 | -0.25 | -0.20 | 0.80 |
| LSI-CoVA-017 | 0.32 | 0.80 | 0.31 | 0.43 | -0.51 | -0.26 |

**Supplementary Table 3: Determination of Spike:LSI-CoVA-017 binding stoichiometry**

The absolute quantities of Spike (purified from mammalian cell culture), heavy and light chains of LSI-CoVA-017 were estimated based on the SDS-PAGE band intensities relative to quantification standards. The quantities of LSI-CoVA-017 were calculated based on the quantities of either heavy chain or light chain. Both approaches resulted in similar quantities determined of LSI-CoVA-017, and thus consistently suggest a binding stoichiometry of three LSI-CoVA-017 bound per Spike trimer. To confirm the reliability of the densitometry, samples were loaded at two different amounts and analyzed from each peak of size-exclusion chromatogram.

| **Peak A (40 µl)** | **mg** | **MW(kDa)** | **pmol** | **Spike trimer/IgG (pmol)** | **LSI-CoVA-017:Spike trimer stoichiometry** |  |
| --- | --- | --- | --- | --- | --- | --- |
| Spike | 3.02 | 160.00 | 18.88 | 6.29 |  |  |
| Heavy Chain | 1.87 | 50.00 | 37.33 | 18.67 | 2.96 |  |
| Light Chain | 0.95 | 25.00 | 37.87 | 18.93 | 3.01 |  |
| **Peak A (25 µl)** | **mg** | **MW(kDa)** | **pmol** | **Spike trimer or IgG (pmol)** | **LSI-CoVA-017:Spike trimer stoichiometry** |  |
| Spike | 2.02 | 160.00 | 12.63 | 4.21 |  | |
| Heavy Chain | 1.23 | 50.00 | 24.53 | 12.27 | 2.91 |  |
| Light Chain | 0.61 | 25.00 | 24.53 | 12.27 | 2.91 |  |
| **Peak B (100 µl)** | **mg** | **MW(kDa)** | **pmol** | **Spike trimer or IgG (pmol)** | **LSI-CoVA-017:Spike trimer stoichiometry** |  |
| Spike | 2.53 | 160.00 | 15.81 | 5.27 |  |  |
| Heavy Chain | 1.57 | 50.00 | 31.33 | 15.67 | 2.97 |  |
| Light Chain | 0.82 | 25.00 | 32.67 | 16.33 | 3.09 |  |
| **Peak B (50 µl)** | **mg** | **MW(kDa)** | **pmol** | **Spike trimer or IgG (pmol)** | **LSI-CoVA-017:Spike trimer stoichiometry** |  |
| Spike | 1.30 | 160.00 | 8.13 | 2.71 |  |  |
| Heavy Chain | 0.81 | 50.00 | 16.13 | 8.07 | 2.98 |  |
| Light Chain | 0.41 | 25.00 | 16.53 | 8.27 | 3.05 |  |

**Supplementary Table 4: Monitoring dynamics of antigen-antibody complexes**

Details of simulation setup for molecular dynamics of top 5 models of complexes between RBD/NTD and Fab domains of LSI-CoVA-014, LSI-CoVA-015, LSI-CoVA-016 and LSI-CoVA-017, as described in Methods. Models highlighted in bold show simulations performed in triplicates.

| **System** | **Model#** | **No. of water ions** | **No. of Na^+^ ions** | **No. of Cl^-^ ions** | **Box size**  **nm × nm × nm** | **Production run (ns)** |
| --- | --- | --- | --- | --- | --- | --- |
| **LSI-CoVA-014-RBD** | Model 1 | 71807 | 203 | 212 | 13.4×13.4×13.4 | 200 |
|  | **Model 2** | **64934** | **185** | **194** | **13×13×13** | **200 × 3** |
|  | Model 3 | 87998 | 250 | 259 | 14.3×14.3×14.3 | 200 |
|  | Model 4 | 73478 | 208 | 217 | 13.5×13.5×13.5 | 200 |
|  | Model 5 | 77021 | 218 | 227 | 13.7×13.7×13.7 | 200 |
| **LSI-CoVA-015-RBD** | **Model 1** | **95807** | **273** | **283** | **14.7×14.7×14.7** | **200 × 3** |
|  | Model 2 | 78446 | 223 | 233 | 13.8×13.8×13.8 | 200 |
|  | Model 3 | 85899 | 245 | 255 | 14.2×14.2×14.2 | 200 |
|  | Model 4 | 57277 | 162 | 172 | 12.5×12.5×12.5 | 200 |
|  | Model 5 | 57277 | 163 | 173 | 12.5×12.5×12.5 | 200 |
| **LSI-CoVA-016-RBD** | Model 1 | 76917 | 218 | 227 | 13.7×13.7×13.7 | 200 |
|  | Model 2 | 80876 | 229 | 238 | 13.9×13.9×13.9 | 200 |
|  | Model 3 | 82268 | 234 | 243 | 14×14×14 | 200 |
|  | **Model 4** | **85898** | **245** | **254** | **14.2×14.2×14.2** | **200 × 3** |
|  | Model 5 | 85946 | 245 | 254 | 14.2×14.2×14.2 | 200 |
| **LSI-CoVA-017-NTD** | Model 1 | 129534 | 368 | 372 | 16.2×16.2×16.2 | 200 |
|  | Model 2 | 110527 | 314 | 318 | 15.4×15.4×15.4 | 200 |
|  | **Model 3** | **85427** | **243** | **247** | **14.2×14.2×14.2** | **200 × 3** |
|  | Model 4 | 115354 | 327 | 331 | 15.6×15.6×15.6 | 200 |
|  | Model 5 | 78027 | 222 | 226 | 13.8×13.8×13.8 | 200 |

**Supplementary Table 5: Primers used to generate mutants**

A list of primers used for generating corresponding mutation is tabulated.

| **Mutation** | **Primer** |
| --- | --- |
| L452R F | AGGTATAGATTGTTTAGGAAGTCTAATCTC |
| L452R R | GTAATTATAATTACCACCAACCTTAGAA |
| T478K R | AACACCATTACAAGGTTTGCT |
| E484 F | GAAGGTTTTAATTGTTACTTTCCTTTA |
| D21 R | CAAAAGGGCACAAGTTTGTAATATTAGGAA |
| I14 F | ATTACAAACTTGTGCCCTTTTGATGAAG |
| G230 F | GCACAGGTGTTCTTACTGAGTCTAAC |
| E236 R | CTCAGTAAGAACACCTGTGCCTTTTAA |
| OA67 R | GTTTGTGCCGCTGATGACGTGGAACC |
| OL981 F | CTGTGCTGAACGATATCTTTTCTAGACTGGA |
| OV67 F | GTCATCAGCGGCACAAACGGAACC |
| OL981 R | AAAGATATCGTTCAGCACAGAGCTGAT |
| PHL HP G682 F | GGGTCTGCTTCCTCTGTAGCTA |
| HP D950N R | GTTCTGCAGTTTGCCCAGGGC |
| HP D950N F | CTGGGCAAACTGCAGAACGTGGTG |
| HP T19R R | AGCTGGGTTCGGGTTCTCAGGTTCAC |
| HP T19R F | AGAACCCGAACCCAGCTCCCACCA |
| HP T95I R | GATACTGGCGAAGTAAACTCCATCATTG |
| HP T95I R1 | AATACTGGCGAAGTAAACTCCATCATTG |
| DHP T95I F1 | GTTTATTTCGCCAGTATTGAGAAGTCCAATATTATTC |
| DHP 156G R | TCCACTTTCCATCCAGCTTTTATTATTCTTATGGTAATACAC |
| DHP 680R R | TACAGAGGAAGCAGACCCCCTTGAGTTAGTTTGTGTC |
| DHP 159V F | AAGCTGGATGGAAAGTGGAGTGTACAGTTCCGCCAACAAC |
| DHP 156G F | GGTGTGTACAGTTCCGCCAACAACTGT |
| DHP 155S R | GGATTCCATCCAGCTTTTATTATTCTTATGGTAA |
| E236 T R | CTCAGTAAGAACACCTGTGCCTGTTAA |
| SHP 14 R | AGTTCTGGTTATAAGATTAACACACTGGCTGCTCACCAGAGGCAG |
| SBA2 15 F | TGTGTTAATCTTATAACCAGAACTCAATCATACACTAA |
| SBA2 966 R | AGCTGATGGCGCCAAATTTGCTGCTGAGCTGCTTCACCA |
| SHP 967 F | TTTGGCGCCATCAGCTCTGTGC |
| SEQ 01B | CGGATCTCTAGCGAATTCC |
| SEQ 03 | CCTTTATTAGCCAGAGGTCG |
